# K–12 Science, Technology, Engineering, and Math characteristics and recommendations based on analyses of teaching cases in China

**DOI:** 10.3389/fpsyg.2022.1010033

**Published:** 2022-09-21

**Authors:** Yunxiang Zheng, Panpan Liu, Xinru Yang, Yidong Guo, Xinxin Qiu, Xiunan Jin, Xianfei Luo, Tianxiang Zheng

**Affiliations:** ^1^School of Educational Information Technology, South China Normal University, Guangzhou, Guangdong, China; ^2^Department of E-Commerce, Jinan University, Shenzhen, Guangdong, China

**Keywords:** STEM education, STEM characteristics, teaching cases, analytical metrics, case analysis

## Abstract

Science, Technology, Engineering, and Math (STEM) education emphasizes solving problems in authentic contexts and developing 21st-century skills. It also helps to cultivate individuals possessing scientific curiosity and innovative abilities. These capacities align with China’s core literacy training. Recent years have seen K–12 STEM cases flourish nationally. However, little attention has been paid to the shared characteristics of these practices, and suggestions for implementing STEM in primary and secondary schools are scarce. This paper presents commonalities in STEM practices within China from a curriculum perspective and offers recommendations for implementation based on these attributes. Specifically, this study first constructed analytical metrics *via* the Delphi method to assess STEM cases. Next, 51 typical STEM teaching cases in different regions of China were analyzed using these metrics. Based upon the statistical results, five characteristics of STEM cases were summarized: China’s STEM education has an unbalanced geographical distribution; current practices benchmark the need for innovative talent training; most instructional content is drawn from real-world problems, but interdisciplinary integration deserves closer focus; the cases featured rich teaching activities and were conducted in a project-based learning fashion with insufficient emphasis on mathematical applications; and China seems to be holistically promoting STEM education, especially through new technologies and supplementary materials. Findings should allow instructors to better understand the intricacies of STEM implementation and to promote successful STEM cases. Recommendations are also provided to optimize the localization of STEM education in China in order to cultivate innovative and interdisciplinary talent.

## Introduction

Science, Technology, Engineering, and Math (STEM) education, a domain conceived by the U.S. National Science Foundation, is a student-centered approach that encourages people to problem solve *via* scientific methods. It emphasizes tackling real-world problems (Honey et al., 2014) using 21st-century skills (e.g., critical and creative thinking, research and questioning, communication, and teamwork) in school settings (Falloon et al., 2020). This type of education prepares students to view issues through an interdisciplinary lens. Students also learn to apply scientific and technical knowledge in multiple life domains and to innovatively solve problems. Ultimately, STEM training enables students to cultivate skills they will need in the future.

STEM education enjoys worldwide popularity; its practices are of great importance within primary, secondary, and higher education (Bolatli and Korucu, 2018; Bryan and Guzey, 2020). STEM is especially useful in K–12 settings—it lays a foundation for a nation’s future science and engineering workforce, prosperity, and even security. Although various views exist regarding the nature of K–12 STEM education, stakeholders have come to focus on STEM integration. The term “integrated STEM” refers to the deliberate combination of core disciplinary content from STEM disciplines (Guzey et al., 2016; Bryan and Guzey, 2020). STEM is no longer considered four isolated disciplines that are implemented individually; the domain is instead treated as a single unified discipline. For the purposes of this paper, “STEM” refers to integrated STEM unless stated otherwise.

Several studies have documented the advantages of integrated STEM education and the benefits of students’ engagement in STEM activities in K–12 classrooms (English, 2017; Means et al., 2017; Kim et al., 2018; Gardner and Tillotson, 2019). Based on these success stories, together with the idea that STEM education can meet scholastic needs for talent training, China’s STEM education has flourished. A growing number of STEM cases have thus emerged nationally, especially in primary and secondary schools. To further advance STEM education, China’s “Ministry of Education of the People’s Republic of China (2022)” proposes fostering students’ interdisciplinary literacy by explaining interdisciplinary concepts based on core aspects of each subject area and then applying these core elements to real situations. Instructors are now encouraged to implement STEM in their teaching. However, involving K–12 students in STEM education calls for reforms to curricula, pedagogy, and the learning environment to ensure a focus on disciplinary knowledge as well as creativity, reasoning, and innovation (Freeman et al., 2019). Most K–12 schools in China, especially those in underdeveloped rural areas, have little experience with such practices (Xu et al., 2021; Wang, 2022). Instructors in these schools may face obstacles when selecting topics, designing activities, and combining related disciplines. To address these concerns, the “China STEM Education Innovation Action Plan 2029” recommends highlighting successful models of STEM education and sharing examples of sound practices nationwide (National Institute of Education Sciences, 2017). Yet sporadic experience cannot be replicated, and a single case only spreads across a small area. A dearth of analysis on the characteristics of K–12 STEM practices in China precludes the smooth development of such education. A literature review spotlighted the lack of attention to common characteristics from a curriculum perspective. Scholars have also rarely offered suggestions for implementing STEM in primary and secondary schools based on these attributes. As such, government officials are unfamiliar with the constitution of STEM education. Instructors in most schools possess a limited sense of what STEM practice entails from a curriculum perspective. These considerations necessitate a systematic analysis of STEM cases to reveal how STEM is implemented.

This paper outlines common STEM practices in China from a curriculum perspective and offers corresponding guidance on implementation. Findings are expected to help instructors better understand how to implement STEM, to promote success in this area, and to foster the localization of STEM education throughout the country. It is first necessary to determine how to evaluate K–12 STEM practices from an instructional design standpoint. To date, relevant studies in China have either covered individual STEM projects or assumed a non-curriculum view. Suitable analytical metrics must therefore be developed. These metrics will lay the groundwork for case analysis and relevant recommendations. It is similarly necessary to discuss typical STEM cases and discern their commonalities based on these metrics. The results of this endeavor are expected to inform suggestions for ways to incorporate STEM into the school curriculum and implement it through teaching reform to promote its localization in China.

The following questions drive this research:

**RQ1:** How can a K–12 STEM case be systematically analyzed from a curriculum perspective?
**RQ2:** What characteristics do typical STEM cases in China share?
**RQ3:** What suggestions can be made for implementing STEM in primary and secondary schools in China based on these characteristics?

## Related work

### Cross-case analysis or evaluation of K–12 STEM practices

Cross-case analysis or evaluation of K–12 STEM practices has garnered extensive interest. For example, STEMworks at Wested (Stemworks at Wested, 2017) proposed a rubric for STEM projects that aligns with a set of common “Design Principles for Effective STEM Philanthropy.” The group sought to create a framework for corporate engagement that improves youth’s STEM performance. Lynch et al. (2018) conducted a cross-case analysis of case studies describing the design and implementation of eight “exemplar” inclusive STEM high schools. The authors identified several critical components and painted a picture of how these high schools achieved their goals (e.g., administrative structure, college-preparatory STEM-focused curriculum, well-prepared STEM teachers). Falloon et al. (2021) performed a cross-case analysis to evaluate factors contributing to the development of four contrasting schools’ STEM profiles. The schools were found to have delivered STEM curricula that met students’ learning needs in the local context. Guzey et al. (2016) judged 20 STEM integration units using the STEM Integration Curriculum Assessment tool and compared them. Łukaszczyk and Grebski (2020) carried out a comparative analysis of the curriculum by selecting two STEM-oriented high schools in Poland and the United States.

In China, Li and Xu (2018) scrutinized eight outstanding science, technology, engineering, arts, and mathematics (STEAM) education cases in the United States. Their analysis addressed educational goals, characteristics, practice, and evaluation. Results indicated that STEAM education can uniquely nurture innovative talent by integrating art and STEM. Wang et al. (2021) considered 45 typical STEM curriculum cases in China and the United States to explore trends in both countries and put forward actionable suggestions for optimal STEM curricula in China. Wang et al. (2019) examined eight typical STEM education programs in the United States from four dimensions: evaluation subject, evaluation object, evaluation content/indicator, and supervision and feedback. The authors discovered that American STEM education displayed a relatively complete closed-loop evaluation mechanism. Chen K. et al. (2021) conducted content analysis on 78 STEM teaching cases in major journals in the fields of physics, chemistry, biology, and geography. They observed clear disciplinary attributes of STEM instructional design in middle schools; however, the use of some key pedagogical strategies was lacking, and a detailed evaluation instrument was absent. Several master’s students (Miao, 2018; Yin, 2019; Lv, 2020; Qiao, 2020) respectively analyzed prototypical STEM courses from the United States and China.

### Characteristics of K–12 STEM practices

Many researchers have examined the attributes of STEM practices. Hansen (2014) generated value-added estimates in math and science to categorize schools into performance levels and evaluated differences in school-attributed STEM outcomes using longitudinal data on students in the United States. states of Florida and North Carolina. Scott (2009) considered 10 STEM-focused high schools in the United States and identified key design components. Schools were selected from various regions across the country. He noted that half of the high schools used a lottery system to select students; in addition to coursework requirements, students also needed to complete internships and/or a capstone project. At the curriculum level, Okulu and Oguz-Unver (2021) developed a measure to evaluate STEM activities using a case study method. The STEM activity assessment form was developed based on a literature review and experts’ opinions. Nite et al. (2017) explicated the characteristics of STEM teaching and learning in middle and high school and in informal settings by examining 58 research sources between 2005 and 2012. Associated themes included reform-based teaching and learning, informal education, teacher factors, and technology use. Sources in different categories were compared based on their features.

As described, limited research has entailed STEM analysis. Non-Chinese scholars appear especially interested in the holistic assessment of STEM projects. They have especially focused on the return on investment, which can help schools and governments more thoroughly evaluate the impact of STEM in K–12 education. Chinese scholars have tended to compare STEM cases from different countries and to investigate the teaching modes or instructional design of these curricula. Far less is known about the shared elements of these practices from a curriculum perspective. Scholars have also rarely offered suggestions for implementing STEM in primary and secondary schools based on these attributes. Given the scope of STEM practices throughout China, a closer analysis of several cases can highlight typical characteristics at the curriculum level. Findings are intended to inform teachers’ design, implementation, and refinement of local STEM courses.

## Methodology

Similar to prior work (Carter, 2013; Hansen, 2014; Okulu and Oguz-Unver, 2021), metrics were created in this study based on a literature review and experts’ opinions when analyzing selected cases. Accordingly, to address the three questions raised in Section “Introduction,” we first constructed a set of analytical metrics. We next chose several cases for fine-grained investigation using the designed metrics and distilled cases’ common features. Then, we identified areas for improvement.

### Instrumentation

To answer RQ1, we extracted core elements of a K–12 course based on China’s Primary and Secondary School Curriculum Standard, elicited key aspects of STEM, and compared several STEM design models *via* a literature review. Quantitative content analysis was used to clarify the items of interest for these cases, from which primary indicators were obtained. We then identified secondary indicators to construct preliminary analytical metrics. Next, the metrics were finalized *via* the Delphi method (Rowe and Wright, 1999; Green, 2014): a team of experts, researchers, and front-line teachers in STEM were invited to assess the metrics’ rationality and give suggestions for revision. The Delphi technique is grounded in a series of questionnaires and iterations. The first survey may include general questions. In each subsequent stage, the questions become more specific in relation to responses on previous questions. Questionnaires in this study were distributed *via* email for experts to complete (and offer feedback) to ensure participant anonymity. We, as the researchers, integrated their comments and then quantitatively interpreted the collected data. The expert panel was given a summary of expert judgments and then pondered their opinions in light of this information. A consensus was reached after two rounds of iteration. The resultant metrics consisted of six primary indicators (background information, teaching objectives, knowledge content, teaching activities, teaching support, and teaching evaluations) and 30 secondary indicators (see Section “Analytical metrics” for details).

To resolve RQ2, we selected and analyzed several STEM cases in China based on our metrics. Secondary indicators related to background information and teaching evaluations were measured qualitatively through content analysis and quantitatively *via* statistics (e.g., average, standard deviation, word frequency). The other indicators were measured quantitatively on 5-point Likert-type scales ranging from 1 (*non-compliant*) to 5 (*completely compliant*). We discerned commonalities across these sample cases. Finally, we made several recommendations for improvement when implementing STEM in primary and secondary schools in China by combining the above attributes with national conditions; in doing so, we responded to RQ3.

### Sampling

Each profiled K–12 STEM teaching case met several criteria. First, each case was implemented using project-based learning (PjBL); that is, it was independently carried out in line with the PjBL principles of planning, creating, processing, and evaluating. Second, each case was interdisciplinary (i.e., it conformed to the characteristics of STEM education and was distinguishable from general teaching in its interdisciplinary nature). Third, each case was whole-class-oriented—teaching objects were students from a whole class in a primary or secondary school (vs. small groups in a school club). Fourth, each case had recently been completed (i.e., between 2018 and 2021) to provide a timely sense of China’s STEM practices. Fifth, each case featured abundant resource support: rich materials were available for analysis (e.g., instructional handouts, photos, or videos; archives of student worksheets or other student work). Our formal dataset consisted of 51 K–12 STEM teaching cases in China.

### Analysis procedure

To improve the accuracy and objectivity of our results, several analysts were asked to review the same STEM teaching case. Results were subjected to a Kappa test for consistency (*via* SPSS). Analysts appeared to agree in their case judgments (Kappa = 0.679). The analysts later shared their opinions of the cases, resolved disagreements through discussion, and evaluated the 51 cases according to the established metrics. Results of the analysis were visualized through radar charts and bar charts in Excel. Some indicators (e.g., topics and keywords) were further imported into SmartAnalyze to build word cloud diagrams. The characteristics of all STEM cases were summarized to devise corresponding suggestions.

## Analytical metrics

### Theoretical background

#### Core elements of a K–12 course

According to China’s Primary and Secondary School Curriculum Standard, each K–12 course includes a purpose, objectives, content, and implementation. These core elements were deconstructed into indicators or sub-indicators for analysis.

#### Key features of STEM

The “White Paper on STEM Education in China,” issued by the National Institute of Education Sciences (2017), frames science and technology innovation education as a lifelong learning activity. STEM is an interdisciplinary and interprofessional domain that serves as a carrier for inclusive student training. This type of education also requires joint participation from society to realize educational innovation, exemplifying STEM’s interdisciplinary nature.

Besides interdisciplinarity, STEM education partly relies on PjBL; that is, presented problems are usually authentic based on the curriculum. Associated PjBL activities include the presentation of context (importing), problem identification, group-based problem exploration, group-based manual engineering, displays of achievement, and self-reflection. Put simply, projects require students to conduct research similarly to a scientist, test with technology like an engineer, and think as a mathematician would. PjBL dominates STEM learning models (Khotimah et al., 2021). The features of interdisciplinarity and PjBL should thus be captured in an analytic model of STEM teaching cases. Kang (2019) noticed that more than half of all students, both elementary and secondary, identified either subject integration or group work as the most distinctive feature of STEAM classes. This pattern further supports the above two attributes.

#### STEM design models

Li and Li (2019) argued that a STEM curriculum includes four basic elements: the STEM topic, learning objectives, learning activities, and supporting materials. Yu and Hu (2015) built a STEM design model based on constructivism; it comprises teaching analysis, a learning task, learning scaffolds, learning activities, tools and resources, learning evaluations, summary and exercises, and experimentation and improvement. Learning objectives, theme selection, learning activities, teaching evaluations, and learning support should hence be considered when designing a STEM project.

To provide authentic learning settings and to enable students to make connections among STEM disciplines, teachers should provide interdisciplinary activities to engage students rather than merely lecturing to impart knowledge. Such activities are integral to STEM education—they shape how students learn and communicate. These activities also inform students’ thinking abilities, collaboration skills, presentation skills, and problem-solving approaches. Common STEM activity models include the 5E inquiry STEM teaching model (Zhao et al., 2018), the 6E learning design model (Barry, 2014), and the 5EX design model (Li and Li, 2019). These models’ main components are listed in Table 1; as indicated, engagement, exploration, design and explanation, and engineering evaluation should be considered when designing STEM activities.

**Table 1:** Components of three STEM activity models.

**Model**	**Components**
5E	Engagement, Exploration, Explanation, Elaboration, Evaluation
6E	Engage, Explore, Explain, Engineer, Enrich, Evaluate
5EX	EQ (Scenario Entering and Question Raising), EM (Scientific Exploration and Mathematical Application), ET (Engineering Design and Technical Making), EC (Knowledge Expansion and Creative Design), ER (Multi-evaluation and Learning Reflection)

### Construction of analytical metrics

#### Analysis items for STEM cases

By referencing the scoring criteria for 2021 outstanding national cases of STEM education in primary and secondary schools, we examined 13 indicator systems (i.e., models/frameworks) related to STEM evaluation (National Research Council, 2009; Change the Equation, 2014; Maryland State Department of Education, 2014; Guzey et al., 2016; Holmlund et al., 2018; Li and Xu, 2018; Miao, 2018; Yin, 2019; Lv, 2020; Qiao, 2020; Lakanukan et al., 2021; Wang et al., 2021). The systems are summarized in Table 2 (items with a frequency of <3 were omitted). Items such as content, evaluation, objectives, implementation, and cooperation appeared frequently and were adopted to construct analytical metrics.

**Table 2:** Ranking of relevant items for STEM evaluation.

**Item**	**Frequency**	**Item**	**Frequency**
Content	13	Design	4
Teaching	12	Processes	4
Course	11	Method	4
Student	11	Skills	4
Evaluation	9	Technology	3
Objectives	9	Explore	3
Implementation	8	Theme	3
Activities	7	Strategy	3
Resources	6		
Cooperation	6		

Each case included background information such as the number of students, number of class hours per week, and related subjects (respectively falling under the indicators of “teaching,” “student,” and “implementation” in Table 2). We combined several items and extracted six for STEM case analysis: (1) background information (covering “teaching,” “course,” “student,” and “implementation”); (2) teaching objectives (covering “objectives”); (3) knowledge content (covering “content”); (4) learning activities (covering “activities” and “cooperation”); (5) teaching evaluations (covering “evaluation”); and (6) supporting materials (covering “resources”). These categories account for the first 10 items in Table 2 and served as primary indicators among our analytical metrics. Specifically, a case’s background information reflects teaching objectives, based on which appropriate STEM content can be chosen. Learning activities can then be designed accordingly. Teaching evaluations convey whether teaching objectives have been achieved. Tools, technology, and resources (common supporting materials) facilitate STEM teaching. These six items were confirmed as primary indicators among our metrics.

#### Preliminary metrics and optimization

We created several sub-indicators (also called “secondary indicators”) to explore the chosen cases. In accordance with Sections “Theoretical background” and “Key features of STEM,” and coupled with relevant studies (Clarke, 2015; Jiang and Cai, 2017; Lin, 2017; National Institute of Education Sciences, 2017; Shaw, 2018; Kang, 2019; Office of Educational Technology, 2019; Durovic, 2020; Falloon et al., 2020; Geesa et al., 2021; Lakanukan et al., 2021; Sirajudin and Suratno, 2021), we decomposed the six primary indicators into secondary indicators as displayed in Table 3.

**Table 3:** Decomposition of six primary indicators.

**Primary Indicators**	**Secondary Indicators**	**Description**
Background Information	Topic selection	Options include validating, exploring, designing, manufacturing, and creating
	Related subjects	Options: Chinese, math, English, science, physics, chemistry, biology, geography, information technology, music, arts, history
	Class hours	Number of teaching hours per week and total number of teaching hours
	Grade	Students’ grade(s)
	Class size	Number of students per class
Teaching Objectives	Interdisciplinary knowledge and skills	Students master basic principles and skills to solve problems in an interdisciplinary manner
	Scientific spirit	Students can think rationally; raise and analyze questions; and solve problems by formulating hypotheses, exploring, and interpreting data to draw conclusions
	Innovative ability	Students can develop innovative solutions or optimize existing solutions using technology
	Cooperative spirit and ability	Students work in teams to communicate, collaborate, and share with others
Knowledge Content	Real-world scenario	Problems relate to real life
	Conforms to curriculum standard	Knowledge content conforms to the curriculum standard and students’ cognitive level
	Interdisciplinary integration	The content of each associated subject is well integrated
	Targeted content	Content is well organized and topic-specific
Learning Activities	Real situation introduction	Teacher demonstrates real-world problems for students from the outset
	Scientific exploration	Teacher encourages students to think rationally, pose questions, and solve them by formulating hypotheses and presenting/evaluating evidence to engage in scientific argumentation
	Handcrafting with technology	Teacher encourages students to choose appropriate technology/tools/materials to complete their work in a hands-on way
	Engineering design	Teacher helps students define engineering tasks and encourages them to complete tasks like an engineer: by drafting, assembling, testing, and optimizing
	Math application	Teacher encourages students to measure, collect, and analyze data to describe the objective world in a mathematical way
	Creative expansion	Teacher encourages students to improve their work creatively according to practical needs
	Evaluation and reflection	Teacher uses multiple evaluation methods to test the learning effect and encourages students to engage in self-reflection
Supporting Materials	Software	Software required for learning (e.g., programming tools, drawing tools)
	Hardware	Hardware that supports students’ cooperative work (e.g., hammer, wooden slats, scissors, robots)
	Multimedia resources	Multimedia resources to facilitate teaching and learning (e.g., PowerPoint, micro-video, reading material, animation)
	Manual or instruction	Operation manual or activity instruction that guides students through group tasks or participation in self-regulated learning
	Learning logs	Records of how students conduct their learning process
	Evaluation tools	Tools that help teacher and students complete individual or collaborative assessments
Teaching Evaluations	Diagnostic evaluation	Records of students’ existing knowledge and skills gained through pre-test(s)
	Formative evaluation	Records of how students conduct their learning process as evidenced by classroom observations, worksheet assessments, self-reflection reports, and peer-review reports
	Summative evaluation	Tests, quizzes, or other criterion-referenced assessments where a score is assigned based on learner-supplied evidence of having mastered desired knowledge or skills

We adopted the Delphi method to verify the scientific soundness and feasibility of our analytical metrics. Specifically, we designed a consultation form and invited 10 experts in the field to complete it *via* email. They were asked to give their opinions on indicators’ rationality and language. In each round, experts considered every indicator (primary and secondary) separately. If an expert deemed an indicator reasonable, it was assigned a score of 1; otherwise, it was assigned a score of 0 with suggested amendments. Experts returned their feedback *via* email. We subsequently calculated each indicator’s mean and standard deviation, integrated experts’ recommended modifications, and updated the indicators. We then distributed another questionnaire to gather additional feedback on issues up for debate. The metrics were optimized during this repetitive process. In brief, experts’ opinions revolved around the following points:

1. The wording of indicators: for example, “topic selection” was replaced with “topic types,” and the expression of primary indicators was unified.
2. The overlap between certain indicators: for instance, the secondary indicator “evaluation and reflection” overlapped with the primary indicator “teaching evaluations”; the secondary indicators “real-world scenario” and “real situation introduction,” which initially appeared under different primary indicators, shared content.
3. The scope of related subjects: experts pointed out that STEM education could cover all subjects in primary school, including comprehensive practice, ethics, and the rule of law.
4. The consideration of the implementation effects of teaching cases: experts suggested including interviews in the analysis to clarify teachers’ perceptions of their instruction and what students gained.
5. Missing secondary indicators: for example, “venue support” should be considered in STEM practice—many cases capitalized on local resources (e.g., Dan et al., 2017) to add a humanistic slant or used smart classrooms to offer students opportunities to learn by integrating new technologies.

#### Finalized metrics

After two rounds of iteration, the indicators were approved by all experts. We then finalized the analytical metrics (six primary indicators and 30 secondary indicators as presented in Table 4). Weights were not assigned to each indicator; our intention was not to evaluate the advantages and drawbacks of teaching cases but instead to synthetize their characteristics.

**Table 4:** Finalized metrics for STEM teaching cases.

**Primary indicators**	**Secondary indicators**	**Description**
Background information	Topic types	Options: validating, exploring, designing, manufacturing, and creating
	Related subjects	Options: Chinese, math, English, science, physics, chemistry, biology, geography, information technology, music, arts, history, ethics and the rule of law, comprehensive practice
	Class hours	Number of teaching hours per week and total number of teaching hours
	Grade of students	Students’ grade(s)
	Class size	Number of students per class
Teaching objectives	Interdisciplinary knowledge and skills	Students master basic principles and skills of how to solve problems in an interdisciplinary manner
	Scientific spirit	Students can think rationally, raise and analyze questions, and solve problems by formulating hypotheses, exploring, and interpreting data to summarize knowledge
	Innovative ability	Students can develop innovative solutions or to optimize existing solutions using technology
	Cooperative spirit and ability	Students work in teams to communicate, collaborate, and share with others
Knowledge content	Based on real-world problems	Problems originate from the objective world and are related to real life
	Conforms to curriculum standard	Knowledge content conforms to the curriculum standard and students’ cognitive level
	Interdisciplinary integration	The content of each associated subject is well integrated
	Targeted content	Content is well organized and topic-specific
Teaching activities	Scenario startup	Teacher illustrates a scenario at the beginning of the lesson
	Scientific exploration	Teacher encourages students to think rationally, pose questions, and solve them by formulating hypotheses and presenting/evaluating evidence to engage in scientific argumentation
	Handcrafting with technology	Teacher encourages students to choose appropriate technology/tools/materials to complete their work in a hands-on way
	Engineering design	Teacher helps students define engineering tasks and encourages them to complete tasks like an engineer: by drafting, assembling, testing, and optimizing
	Math application	Teacher encourages students to measure, collect, and analyze data to describe the objective world in a mathematical way
	Creative expansion	Teacher encourages students to improve their work creatively according to practical needs
	Presentation and reflection	Teacher encourages students to make presentation to share their work in public and engage in self-reflection.
Teaching support	Software	Software required for learning (e.g., programming tools, drawing tools)
	Hardware	Hardware that supports students’ cooperative work (e.g., hammer, wooden slats, scissors, robots)
	Venue support	On-campus and/or off-campus venues that support teaching and learning
	Multimedia resources	Multimedia resources to facilitate teaching and learning (e.g., PowerPoint, micro-video, reading material, animation)
	Manual or instruction	Operation manual or activity instruction that guides students through group tasks or participation in self-regulated learning
	Learning logs	Records of how students conduct their learning process
	Evaluation tools	Tools that help teacher and students complete individual or collaborative assessments
Teaching evaluations	Diagnostic evaluation	Records of students’ existing knowledge and skills gained through pre-test(s)
	Formative evaluation	Records of how students conduct their learning process as evidenced by classroom observations, worksheet assessments, self-reflection reports, and peer-review reports
	Summative evaluation	Tests, quizzes, or other criterion-referenced assessments where a score is assigned based on learner-supplied evidence of having mastered desired knowledge or skills

## Analysis results

### Regional distribution

**Figure 1:**
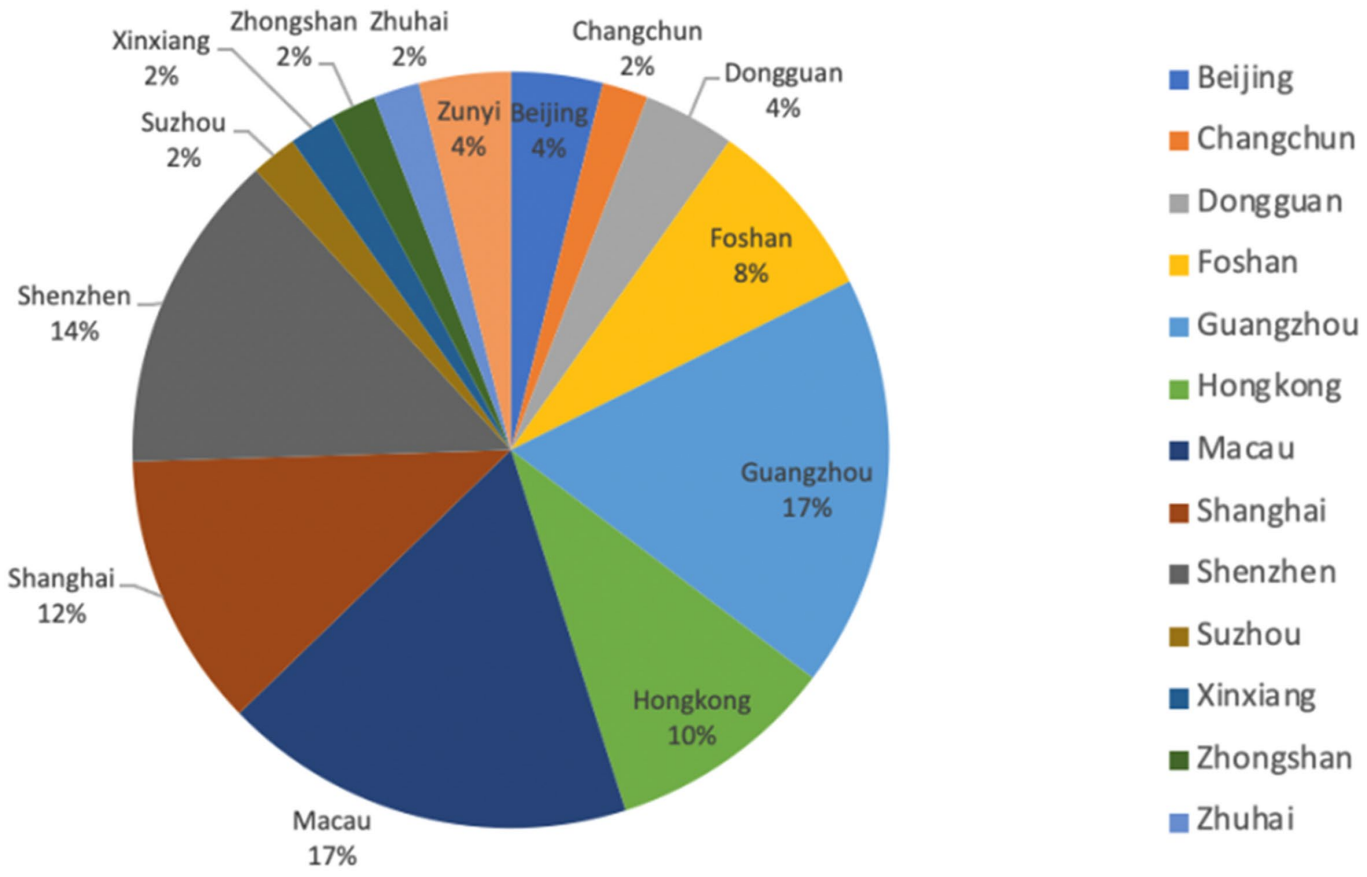
Regional distribution of sample cases.

Figure 1 indicates that the 51 STEM teaching cases spanned 13 regions including Hong Kong, Macau, Shanghai, Beijing, Guangzhou, Zhuhai, and others. Most cases were based in Guangzhou (17%), Macau (17%), Shenzhen (14%), and Shanghai (12%), which is proportional to these regions’ economic power.

### Background information

#### Topic types and topic keywords

**Figure 2:**
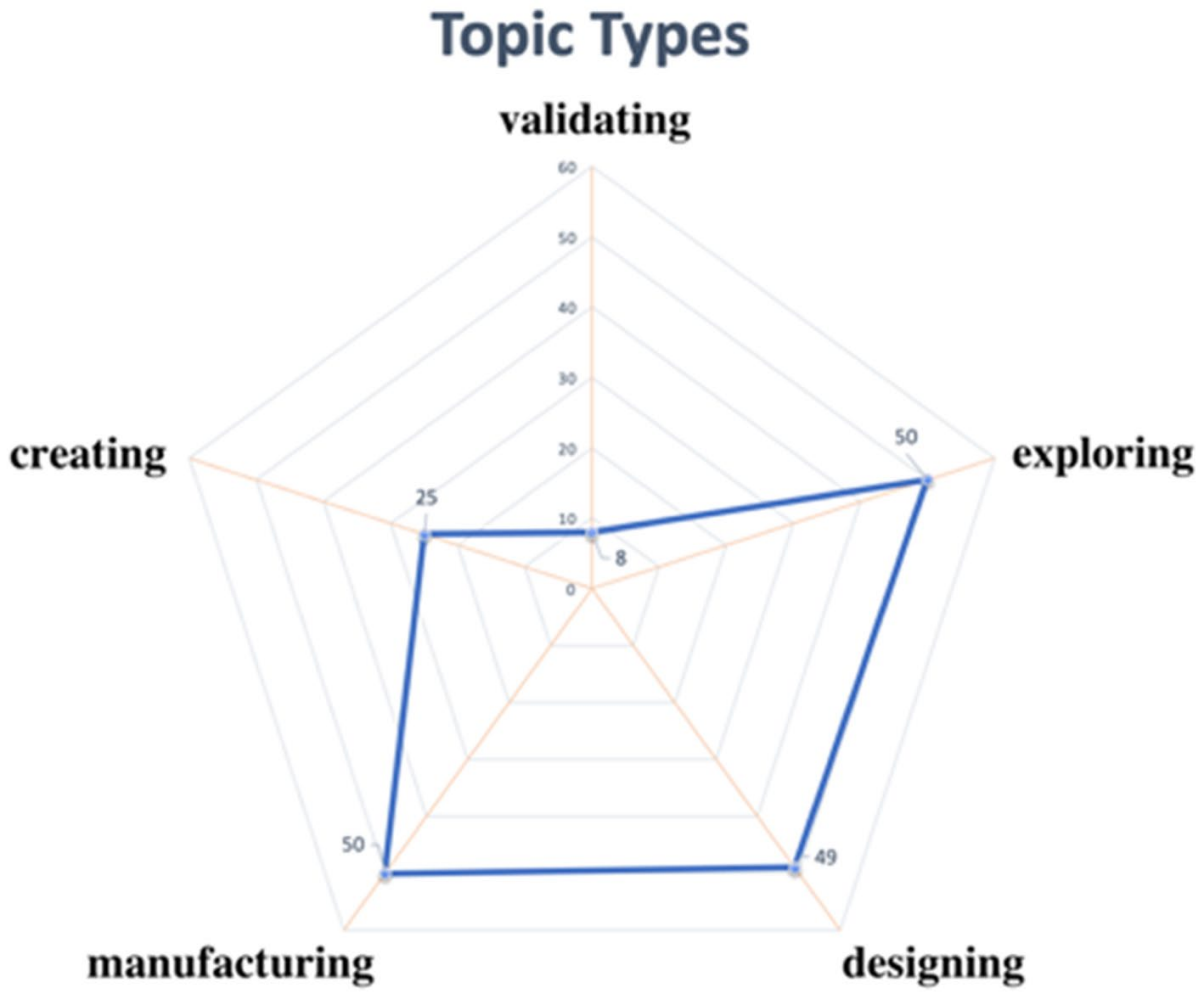
Topic types from sample cases.

**Figure 3:**
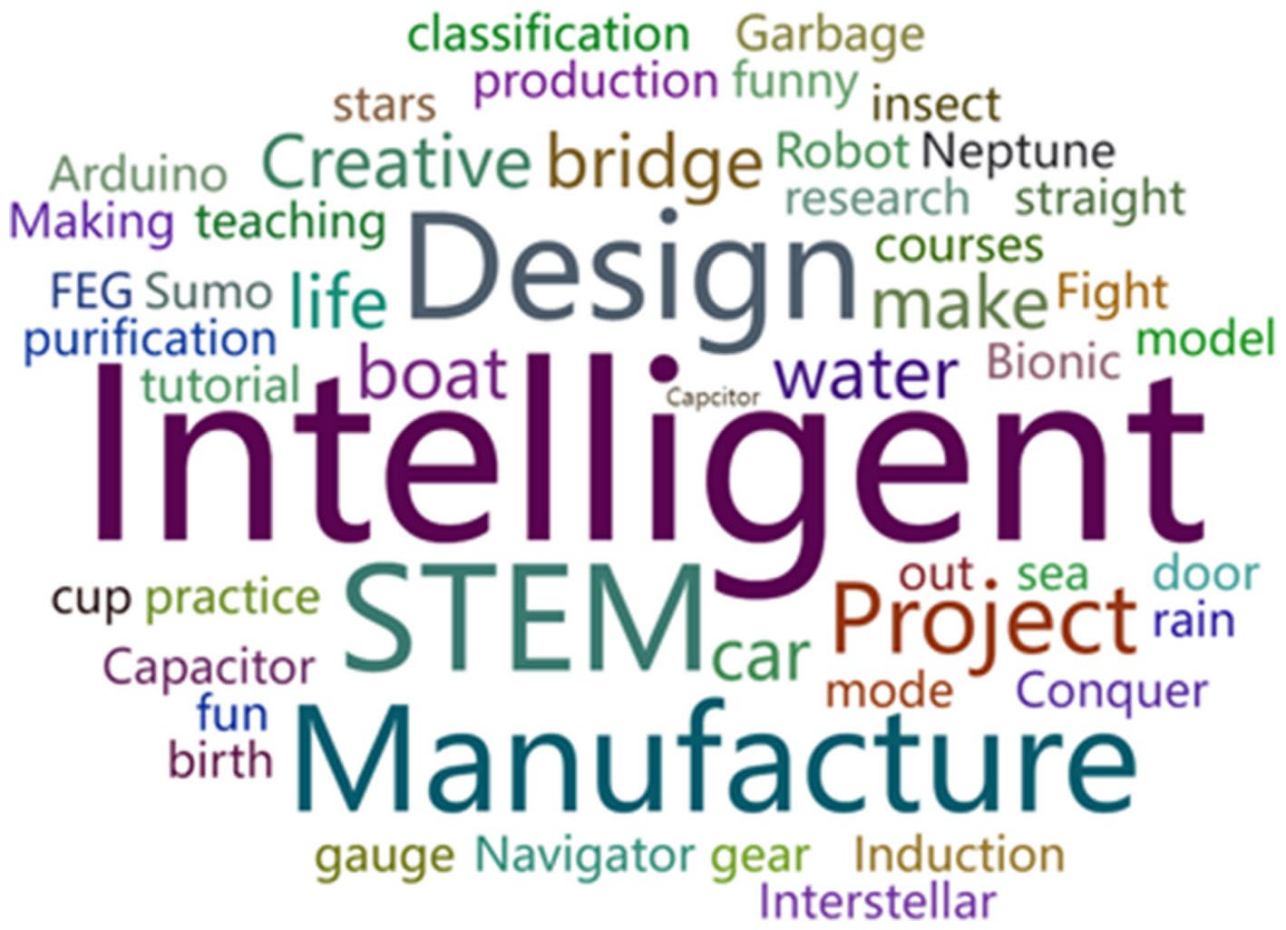
Topic keywords from sample cases.

Figure 2 indicates “exploring,” “manufacturing,” and “designing” as main topic types. The word cloud diagram in Figure 3 shows that “intelligent,” “design,” and “manufacture” were frequently used in naming cases, which mirrors the topic-based keywords. For instance, “Design and Manufacture of Intelligent Nursery,” “Design and Manufacture of Intelligent Fire Alarm,” and “Design of Intelligent Catapult” were popular STEM case names; all originated from real life and have practical significance.

#### Related subjects and class hours

Interdisciplinarity is the cornerstone of STEM. Of the sample cases, each was related to an average of 4.3 subjects, with “math,” “science,” and “physics” accounting for the highest proportion. The most common combination was “math,” “science,” “physics,” and “information technology”; 21 (out of 51) cases fell into this category. In terms of class hours, the cases were generally carried out over one semester, with an average of 15.2 h per case (for a rough duration of 11 weeks when holding two classes per week).

#### Teaching objects

The statistical results demonstrated that these STEM cases were mainly designed and implemented for students in Grades 4–7, with 31 students per class on average.

### Teaching objectives

According to Table 5, the overall objectives of these STEM teaching cases were appropriate, but imbalances persisted: high scores accompanied “innovative practical ability” and “cooperative spirit and ability,” whereas the other two indicators earned relatively low scores. As such, teachers paid more attention to students’ innovative and cooperative abilities than to interdisciplinarity and a scientific spirit. Realization of the interdisciplinary objective was not as noteworthy as anticipated despite each case relating to 4.3 subjects on average.

**Table 5:** Teaching objectives in sample cases.

**Teaching objectives**	***M***	***SD***
Interdisciplinary knowledge and skills	3.90	0.76
Scientific spirit	4.20	0.66
Innovative practical ability	4.29	0.76
Cooperative spirit and ability	4.33	0.71

### Knowledge content

Table 6 shows that most cases highlighted their intended topics, and relevant knowledge content conformed to students’ curriculum standards.

**Table 6:** Knowledge content in sample cases.

**Knowledge content**	***M***	***SD***
Based on real-world problems	4.16	0.73
Conforms to curriculum standard	4.39	0.57
Interdisciplinary integration	4.06	0.61
Targeted content	4.45	0.61

### Teaching activities

Based on Table 7, the design and implementation of teaching activities were unbalanced. Scores on “handcrafting with technology” and “presentation and reflection” were 4.33 and 4.35, respectively—each relatively high. “Math application” was scored the lowest, indicating that it was not fully applied in some cases.

**Table 7:** Teaching activities in sample cases.

**Teaching activities**	***M***	***SD***
Scenario startup	4.14	0.69
Scientific exploration	4.18	0.68
Handcrafting with technology	4.33	0.65
Engineering design	4.10	0.76
Math application	3.96	0.82
Creative expansion	4.12	0.68
Presentation and reflection	4.35	0.63

### Teaching support

As for STEM teaching support, “hardware” was scored highest (see Table 8), whereas “venue support,” “evaluation tools,” “manual or instructions,” and “learning logs” were scored low. We observed that 3D modeling and 3D printing were used in several cases, with some teachers even leveraging intelligent technologies such as sensors or programming with Arduino. Students were also given various physical materials, such as acrylic plates, motors, batteries, and scissors. Statistical forms or diagrams were widely used to help students record experimental data and conduct further analysis.

**Table 8:** Teaching support in sample cases.

**Teaching support**	***M***	***SD***
Software	4.06	0.83
Hardware	4.24	0.62
Venue support	3.86	0.66
Multimedia resources	4.02	0.62
Manual or instruction	3.94	0.68
Learning logs	3.94	0.76
Evaluation tools	3.90	0.78

### Teaching evaluations

All cases included summative evaluations, and most cases included formative evaluations. Only two cases included diagnostic evaluations. Instructors tended to use teacher evaluations and student self-evaluations, with some also using group evaluations (as an example of multivariate assessment). In most cases, multidimensional evaluation forms were provided to assess students’ learning, including their performance on plan proposals, group discussions, handcrafting, and presentations.

## Discussion and recommendations

The above analysis demonstrates that China’s typical STEM education possesses several common characteristics. First, it presented an uneven geographical distribution: many excellent cases emerged in economically developed areas (e.g., Beijing, Guangzhou, Shanghai), whereas effective STEM education seemed rare in less developed areas. Such education relies heavily on numerous types of hardware and software support, which greatly increases expenses. Economically developed areas therefore tend to outperform less developed areas in STEM education (Xu et al., 2021). Globally, the countries best known for excelling in STEM are those with small populations and a relatively well-developed economy (English, 2019). Our finding is consistent with this trend.

Second, current STEM practices in China benchmark the need for innovative talent training as advocated in China’s Primary and Secondary School Curriculum Standard (2022). Our cases related to more than four subjects per case on average. Secondary indicators such as “interdisciplinary knowledge and skills,” “scientific spirit,” “innovative practical ability,” “cooperative spirit and ability,” and “interdisciplinary integration” contribute to the goal of innovative talent cultivation. We noted high scores on these indicators, revealing that they served the aim of innovative talent training. Many schools in our sample offered STEM courses for students in Grades 4–7, and classes contained 31 students on average. These outcomes aligned with those identified by Batdi et al. (2019) and echoed circumstances in the United States (Committee on Highly Successful Schools or Programs in K-12 STEM Education, N. R. C., 2011).

Third, most instructional content was drawn from real-world problems, but interdisciplinary integration deserves more focus. Most cases in our sample showed adequate content selection. Course material usually stemmed from actual problems, enhancing the student experience and exposing students to realistic scenarios. The YouthInsight survey report (YouthInsight for the Department of Industry, S., Energy and Resources of Australian Government, 2021) came to similar conclusions: 72% of teachers reported feeling very confident in connecting STEM content with real-world applications. Yet in our cases, each discipline became fairly self-contained and independent over time, hampering integration. This consequence corroborates that of a prior study (Chen K. et al., 2021). Previous work (Banilower et al., 2018) documented that 35% of middle schools offered single-discipline science courses for students in Grade 6 while 80% offered single-subject mathematics courses. As an interdisciplinary teaching method, STEM mandates teaching across disciplines using strategies that support knowledge integration *via* authentic tasks. However, when instructors who are accustomed to single-discipline content teach STEM courses cooperatively, they tend not to adhere to top-level interdisciplinary design. Interdisciplinary integration was clearly inadequate in these STEM cases.

Fourth, these cases featured rich teaching activities and were conducted in a PjBL fashion with insufficient emphasis on mathematical applications. PjBL enables students to pursue solutions to problems in the same way that professional scientists do (Office of Educational Technology, 2019). The process normally includes brainstorming, planning, discussing, measuring, assembling, experience sharing, and assessing. As an innovative talent cultivation mode, STEM is typically combined with PjBL, a method with demonstrated efficacy (Lou et al., 2017; Kartini et al., 2021; Lakanukan et al., 2021). As shown in Table 7, the teaching activities in these cases were detailed enough to cover those associated with PjBL. These activities are distinct from traditional instructional methods and are expected to cultivate innovative talent. Among the seven teaching activities in this study, mathematical applications received insufficient consideration: in many cases, teachers preferred to simply inform students of the results instead of inviting students to measure, calculate, or make comparisons on their own. The relative lack of mathematical applications in these activities is contradictory to the premise of STEM education (i.e., in ignoring “M”).

Fifth, China seems to be holistically promoting STEM education, especially through new technologies (e.g., hardware and software support) and supplementary materials such as instruction manuals. Different from Wan et al. (2021), where deficient resources were frequently mentioned, the cases in this study featured a wide range of support when implementing STEM courses (e.g., software, hardware, and multimedia resources). These circumstances coincide with a YouthInsight survey (YouthInsight for the Department of Industry, S., Energy and Resources of Australian Government, 2021) highlighting the websites Teachers Pay Teachers, Scootle, and Khan Academy as the most popular online resources for teachers. The Information Resources Management Association of the United States also stressed the roles of digital resources in promoting STEM literacy (Management Association, I. R, 2018). In addition to hardware and software empowered by new technologies (e.g., robots, 3D printing, sensors), supplementary materials such as learning logs can aid students’ collaborative interdisciplinary learning. Worksheets can help bolster students’ critical thinking skills (Hartini et al., 2020). In a related vein, the newest National Survey of Science & Mathematics Education report from the United States (Banilower et al., 2018) outlined various instructional resources for STEM courses, including supplementary materials (e.g., laboratory handouts). Students can accordingly plan, revise, implement, and test solutions to problems *via* engineering design processes and appropriate support technologies. Assessments are embedded in these courses to solicit students’ reflections on the quality of their explanations, models, or problem solutions, calling for a strong record of their learning processes.

Several recommendations arose from our analysis of STEM teaching cases in primary and secondary schools in China. First, the country should harness the development of STEM education in multiple regions. Underdeveloped regions will otherwise struggle to develop first-class STEM education on a large scale. Quality STEM education requires vast investment: funding, venues with high-tech equipment, and highly qualified teachers. These features exacerbate the financial burden for schools—especially those in second- and third-tier cities. To leverage the development of STEM education in different regions while promoting educational balance, China should seek to support STEM practices in these cities. The government should allocate educational resources to second- and third-tier cities as needed and rearrange the distribution of education to give full play to the impact of STEM education. For example, the government should consider increasing investment and promulgating a post-service training plan for STEM teachers in primary and secondary schools in western China (Chen T. et al., 2021). Teaching reform projects and teaching achievement evaluations related to STEM education could also be created (Xu et al., 2021). Stakeholders could additionally refer to farming culture to realize the localization reform and innovation of rural STEM education (Yang, 2020).

Second, primary and secondary schools should continue teaching STEM based on curriculum standards (especially the version released in 2022) and gradually expand to cover all students in the same grade instead of only one or two classes. As reflected in the STEM2026 report (The U.S. Department of Education, A. I. f. R, 2016) and the white paper on STEM education in China (National Institute of Education Sciences, 2017), STEM education entails lifelong learning. This process can stimulate students’ enthusiasm for scientific exploration and innovation. STEM education offers a way to improve all students’ core literacy instead of selectively nurturing exceptional talent. Limited classes were chosen for pilot studies in most cases, contrary to the above goal. We recommend that primary and secondary schools proceed with STEM instruction based on the most recent curriculum standards and extend the breadth of teaching to cover more students in a given grade. As indicated by our findings (especially Figures 2, 3), nearly all walks of life and most disciplines are linked to STEM. Teachers from numerous disciplines are hence encouraged to join STEM pilot programs (according to Section “Related subjects and class hours,” a STEM practice can be related to four or five subjects). Students from the same grade should be welcome to take part in STEM learning based on diverse topics. For instance, students in Grades 1–4 could concentrate on “smart cars,” those in Grades 4–8 could learn about “smart alerts,” and all others could focus on “sea cleaners.” STEM education would therefore be broadened to involve students beyond Grades 4–7 (Section “Teaching objects”).

Third, schools should enhance interdisciplinarity to achieve integrated STEM. Interdisciplinarity in education encourages learners to make connections between disciplines (i.e., combining knowledge and skills from two or more subjects) when solving complicated problems or explaining complex phenomena. STEM teachers thus need to master interdisciplinary knowledge; communicate and cooperate in top-level design; and integrate multidisciplinary content in instructional design, course implementation, and self-reflection. In light of the current discipline-based curriculum and the fact that many teachers are unfamiliar with creating instructional materials for integrated STEM, strategies for instructor collaboration across subjects should be carefully planned. Engaging teachers in professional development for curriculum design is critical to developing integrated STEM. This course of action can improve integrated STEM education and rectify insufficient interdisciplinarity. Several provinces have started to explore this prospect, such as by establishing teacher alliances (e.g., Guangdong–Hong Kong–Macao Greater Bay Area STEM Education Alliance) or conducting network-based teaching and research (Hu et al., 2021). Online and offline teacher training has emerged as other strategies. These efforts can routinely bring teachers together to improve interdisciplinarity while presenting opportunities for further collaboration.

Fourth, schools should continue strengthening mathematical applications in learning activities to endow students with a rigorous academic attitude. Most sample cases involved mathematics, albeit to a lesser extent than expected. Simple measurement and data recording dominated math activities in low-scoring cases. In reality, mathematical knowledge encompasses measuring, marking (tagging), calculating, matching, grading, and comparing. These activities should be fully utilized in STEM learning (Küçük-Demir and Düzen, 2022) to better engage students in data analysis. Doing so can also popularize mathematical tools and compel students to seek knowledge more rigorously. Their core literacy will likely be reinforced as a result.

Fifth, schools should focus on archiving documentation (i.e., quantitative and qualitative analyses) to support formative evaluation. This type of evaluation possesses a unique advantage in tracking students’ STEM performance (vs. ranking students by level). The availability of evidence is also important. Teachers should retain procedural data as students learn, including answer sheets, design drafts, statistical data, and classroom observation forms. Without this documentation, quantitative or qualitative analysis of teaching effectiveness cannot be objectively obtained. Formative evaluation may be weakened as a result. For instance, Okulu and Oguz-Unver (2021) developed an evaluation form to determine activities’ appropriateness with respect to the nature of STEM education, which has four categories (STEM learning environment, activation of students, STEM content and practices, and connecting STEM). The form was found to be useful for evaluating and improving STEM education based on pedagogical approaches such as PjBL and collaborative learning. Moreover, Fitzallen and Watson (2019) stated that statistics provide a firm foundation for bridging STEM disciplines. This perspective recognizes that building on data gathered through classroom activities in STEM settings can potentially support students in honing their statistical literacy. This skill will be advantageous in other social contexts where people encounter such data. These efforts can enhance students’ comprehensive qualities and core competencies through formative assessment.

## Conclusion

Researchers generally agree about the significance of STEM in K–12 education. Driven by national policies, K–12 STEM education in China has ushered in hundreds of practical cases. Scholars have extensively addressed the effectiveness of applying STEM in K–12 settings. Much less interest has surrounded these practices’ curricular similarities. Suggestions for implementing STEM in primary and secondary schools based on these characteristics are scarce as well. The current study is hoped to enable instructors to better understand STEM implementation, to prompt successful STEM cases, and to promote the localization of STEM education. Common characteristics were discerned using a curriculum perspective, with affiliated suggestions for implementing STEM based on these attributes. We first established analytical metrics (including six primary indicators and 30 secondary indicators) to analyze K–12 STEM cases. Fifty-one typical teaching cases throughout China were next examined based on these metrics. Five characteristics were extracted from the statistical results, complemented by recommendations for promoting STEM education in the country. Findings shed light on STEM implementation, its features, and areas for refinement. These enhancements will help nurture 21^st^-century talent with scientific inquisitiveness and innovative skills. Future research could apply our suggestions to STEM practices to assess their efficacy and refine the techniques as needed. Including additional cases from throughout China could also unearth more meaningful findings.

## Data availability statement

The raw data supporting the conclusions of this article will be made available by the authors, without undue reservation.

## Ethics statement

The studies involving human participants were reviewed and approved by the Academic Committee Office (ACO) of South China Normal University (http://fzghb.scnu.edu.cn/), Guangzhou. Written informed consent from the participants’ legal guardian/next of kin was not required to participate in this study in accordance with the national legislation and the institutional requirements.

## Author contributions

YZ: conceptualization, methodology, writing—original draft preparation, writing—review and editing, supervision. PL: writing—original draft preparation and writing—review and editing. XY, YG, XQ, and XJ: software, data curation, and formal analysis. XL: writing—original draft preparation. TZ: conceptualization, methodology, writing—review and editing, supervision. All authors contributed to the article and approved the submitted version.

## Funding

This research was funded by the Key Project of China Educational Technology Association’s 14th Five-Year Plan under grant no. G021 and Jinan University Shenzhen Campus Funding Program under grant no. JNSZQH2105.

## Conflict of interest

The authors declare that the research was conducted in the absence of any commercial or financial relationships that could be construed as a potential conflict of interest.

## Publisher’s note

All claims expressed in this article are solely those of the authors and do not necessarily represent those of their affiliated organizations, or those of the publisher, the editors and the reviewers. Any product that may be evaluated in this article, or claim that may be made by its manufacturer, is not guaranteed or endorsed by the publisher.
